# Influence of real-world characteristics on outcomes for patients with methicillin-resistant Staphylococcal skin and soft tissue infections: a multi-country medical chart review in Europe

**DOI:** 10.1186/1471-2334-14-476

**Published:** 2014-09-02

**Authors:** Dilip Nathwani, Christian Eckmann, Wendy Lawson, Caitlyn T Solem, Shelby Corman, Jennifer M Stephens, Cynthia Macahilig, Damien Simoneau, Richard Chambers, Jim Z Li, Seema Haider

**Affiliations:** Ninewells Hospital and Medical School, Ward 42, East Block, Dundee, DD19SY UK; Klinikum Peine, Academic Hospital of Medical University Hannover, Virchowstrasse 8 h, D-31226 Peine, Germany; Hammersmith Hospital, Imperial College Healthcare NHS Trust, Du Cane Road, London, W12 0HS UK; Pharmerit International, 4350 East West Highway, Suite 430, Bethesda, MD 20814 USA; Medical Data Analytics, 4 Gatehall Drive, 2nd Floor, Parsippany, NJ 07054 USA; Pfizer International Operations, 23-25 avenue du Dr Lannelongue, Paris, 75668 France; Pfizer Inc, 500 Arcola Road, Collegeville, PA 19426 USA; Pfizer Inc, 10646 Science Center Drive, San Diego, CA 92121 USA; Pfizer Inc, Eastern Point Road, Groton, CT 06340 USA

**Keywords:** IV-to-oral antibiotic switch, Length of stay, Clinical criteria, Antibiotic therapy

## Abstract

**Background:**

Patient-related (demographic/disease) and treatment-related (drug/clinician/hospital) characteristics were evaluated as potential predictors of healthcare resource use and opportunities for early switch (ES) from intravenous (IV)-to-oral methicillin-resistant *Staphylococcus aureus* (MRSA)-active antibiotic therapy and early hospital discharge (ED).

**Methods:**

This retrospective observational medical chart study analyzed patients (across 12 European countries) with microbiologically confirmed MRSA complicated skin and soft tissue infections (cSSTI), ≥3 days of IV anti-MRSA antibiotics during hospitalization (July 1, 2010-June 30, 2011), and discharged alive by July 31, 2011. Logistic/linear regression models evaluated characteristics potentially associated with actual resource use (length of IV therapy, length of hospital stay [LOS], IV-to-oral antibiotic switch), and ES and ED (using literature-based and expert-verified criteria) outcomes.

**Results:**

1542 patients (mean ± SD age 60.8 ± 16.5 years; 61.5% males) were assessed with 81.0% hospitalized for MRSA cSSTI as the primary reason. Several patient demographic, infection, complication, treatment, and hospital characteristics were predictive of length of IV therapy, LOS, IV-to-oral antibiotic switch, or ES and ED opportunities. Outcomes and ES and ED opportunities varied across countries. Length of IV therapy and LOS (r = 0.66, p < 0.0001) and eligibilities for ES and ED (r = 0.44, p < 0.0001) showed relatively strong correlations. IV-to-oral antibiotic switch patients had significantly shorter length of IV therapy (−5.19 days, p < 0.001) and non-significantly shorter LOS (−1.86 days, p > 0.05). Certain patient and treatment characteristics were associated with increased odds of ES (healthcare-associated/ hospital-acquired infection) and ED (patient living arrangements, healthcare-associated/ hospital-acquired infection, initiating MRSA-active treatment 1–2 days post cSSTI index date, existing ED protocol), while other factors decreased the odds of ES (no documented MRSA culture, ≥4 days from admission to cSSTI index date, IV-to-oral switch, IV line infection) and ED (dementia, no documented MRSA culture, initiating MRSA-active treatment ≥3 days post cSSTI index date, existing ES protocol).

**Conclusions:**

Practice patterns and opportunity for further ES and ED were affected by several infection, treatment, hospital, and geographical characteristics, which should be considered in identifying ES and ED opportunities and designing interventions for MRSA cSSTI to reduce IV days and LOS while maintaining the quality of care.

**Electronic supplementary material:**

The online version of this article (doi:10.1186/1471-2334-14-476) contains supplementary material, which is available to authorized users.

## Background

European healthcare systems are under increased economic pressure owing to greater demand for health services despite stable or declining budgets [1]. Hospitalized patients with complicated skin and soft tissue infections (cSSTI) caused by methicillin-resistant *Staphylococcus aureus* (MRSA) are a substantial contributor to this clinical and economic burden [2–4]. The standard treatment option for patients with MRSA cSSTI is intravenous (IV) antibiotic therapy. Patients often remain hospitalized for the duration of treatment although these infections have a relatively low risk of complications, readmissions, or mortality once the patient has been stabilized.

Treatment options are available that allow some of these patients to complete therapy after discharge from the hospital with either outpatient parenteral antibiotic therapy (OPAT) or oral antibiotic therapy, with oral therapy preferred by patients in many settings [5, 6]. Several oral antibiotic therapies with activity against MRSA are available as options for patients with MRSA cSSTI, including clindamycin, linezolid, rifampicin in combination with another active agent, doxycycline, and trimethoprim/sulfamethoxazole; however, the selection of therapy must be guided by local susceptibility data, as MRSA isolate resistance to these oral agents varies [7, 8]. Oral antibiotic therapy selection also should be guided by other properties, such as tolerability, bioavailability, and efficacy in patients with complicated disease.

In the face of decreasing hospital capacity [9] and increasing economic pressure, exploring approaches to optimize care is important. One key antibiotic stewardship strategy (an approach that supports choice of antibiotic therapy, as well as dose, route of administration and treatment duration) that also has the potential to reduce hospital use is to promote IV-to-oral antibiotic switch therapy, which may facilitate hospital discharge [10, 11]. In an era of scarce resources and tightened healthcare budgets, understanding which patient and treatment setting characteristics drive resource use is important; such resource use includes IV antibiotic days and length of hospital stay (LOS), as well as characteristics associated with opportunities to optimize care further whether met (such as patients who were switched from IV-to-oral antibiotic therapy) or unmet (including cases where patients could have been switched from IV-to-oral antibiotic therapy and/or discharged earlier from the hospital and received oral antibiotics or OPAT in an outpatient setting). To address these needs, this study aimed to explore clinical and demographic characteristics and hospital treatment characteristics associated with actual healthcare resource use and unmet opportunities for early switch (ES) from IV-to-oral MRSA-active antibiotic therapy and early discharge from the hospital (ED).

## Methods

### Study population

This is a secondary analysis of a retrospective observational cohort study which systematically collected data from the medical charts of patients from 12 European countries (Austria, the Czech Republic, France, Germany, Greece, Ireland, Italy, Poland, Portugal, Slovakia, Spain, and the United Kingdom). The aim of the study was to describe treatment patterns and healthcare resource use across Europe and identify opportunities for optimizing patient switch from IV-to-oral antibiotics (early switch [ES]) and early discharge (ED) on oral antibiotics or outpatient parenteral antibiotics [12, 13].

Patients meeting the following criteria were identified by study investigators who were hospital-based infectious disease specialists, internal medicine specialists with an infectious disease subspecialty, or medical microbiologists: had microbiologically confirmed MRSA cSSTI (e.g. deep or extensive cellulitis, infected wound or ulcer, major abscess, or other soft tissue infections requiring substantial surgical intervention), received ≥3 days of IV anti-MRSA antibiotics during their hospitalization (between July 1, 2010 and June 30, 2011, inclusive), and had been discharged alive by July 31, 2011. Anti-MRSA antibiotics included, but were not limited to clindamycin, daptomycin, fusidic acid, linezolid, rifampicin, teicoplanin, tigecycline, trimethoprim/sulfamethoxazole, and vancomycin. Patients were excluded from this study if they were treated for the same cSSTI within 3 months of hospitalization; had suspected or proven diabetic foot infections, osteomyelitis, infective endocarditis, meningitis, joint infections, necrotizing soft tissue infections, gangrene, prosthetic joint infection, or prosthetic implant/device infection; had significant concomitant infection at other sites; were immunosuppressed (e.g. diagnosed with hematologic malignancy, neutropenia, or rheumatoid arthritis; were receiving chronic steroids or cancer chemotherapy); were enrolled in another cSSTI-related clinical trial; or were pregnant or lactating.

Patients included in the study were randomly sampled from all patients meeting inclusion/exclusion criteria from each study investigator’s site. Additionally, we non-randomly oversampled patients who received IV-to-oral antibiotic switch therapy to have sufficient sample size for comparisons between patients receiving IV-only and IV-to-oral antibiotic switch MRSA-targeted therapy. The combination of these 2 groups formed the full sample for this study. After checking the antibiotic treatments for all patients in the full sample, we confirmed a subsample of patients who received MRSA-active antibiotics (MRSA treatment sample).

### Ethics

The sponsor of the study was blinded to the study investigators/institutions collecting data, and likewise the study investigators/institutions were blinded to the sponsor; thus specific names of institutions and ethics boards are not provided. Ethics approvals were obtained based on country- and institution-specific requirements for collection of anonymous, de-identified data from medical charts. It was the responsibility of the investigator at each site to have prospective approval of the study protocol, protocol amendments, and other relevant documents from the Institutional Review Board(s) (IRB) and/or Independent Ethics Committee(s) (IEC), when applicable. In most EU countries for observational retrospective studies, only one application was needed; that is, an exemption or waiver granted to one institution is sufficient or adequate to present to other institutions that may require it. Informed consent was NOT required from the patient under an Ethics Board review study exempt status. Copies of IRB/IEC approvals were kept by MDA due to the double-blind nature of the study. To meet specific country compliance requirements some registrations and ethics applications were completed. In Italy, the study was registered within the central AIFA database. In Germany an independent review board approval was coordinated and received by the study sponsor affiliate. In Ireland two hospital ethics board reviews and approvals were received. All correspondence with the IRB/IEC was retained in the respective Investigator’s file and at Medical Data Analytics (MDA). All approval documentation from the IRB/IEC was retained in the respective Investigator’s file and submitted to MDA.

### Data collection

Medical charts were reviewed using a standardized data collection form to collect information regarding patient characteristics and clinical and resource utilization outcomes. Separate data collection forms were also utilized to collect site-level characteristics including hospital beds available and protocols in place for IV antibiotic use and discharge. All data were retrospectively collected. Key patient and site characteristics that were collected are listed in Table 1. In addition, data were collected on patient treatment patterns including length of IV therapy, length of stay, and whether the patient was switched from IV-to-oral antibiotics for treatment of their MRSA cSSTI and also regarding whether patients had met a series of criteria which were used to operationalize ES and ED (described further below).

**Table 1:** **Patient and site characteristics (independent variables) collected from patient charts**

Type of data collected	Pre-specified as clinically relevant (always included in models)	Considered in models only if marginally statistically significant (p < 0.1)
**Patient baseline characteristics**	• Age (later removed), Country,	• Patient living arrangements (for IV-only, IV-to-oral, ES models),
	• Patient living arrangements (for LOS, ED models)	• Employment status
	• Charlson comorbidity index	• History of diabetes
	• IV drug abuse	• History of diabetes with end organ damage
		• History of peripheral vascular disease
		• History of dementia
		• Any MRSA colonization before admission
**Infection/treatment characteristics**	• cSSTI type	• Days from admission to cSSTI index date
	• cSSTI location	• Initial antibiotic therapy was MRSA active
	• cSSTI source	• Time to initiating MRSA-active therapy
	• Days to first MRSA culture	
	• Any surgical procedures for cSSTI	
	• IV-to-oral antibiotic switch (vs IV-only; except for IV-to-oral antibiotic switch model)	
**Complications**		• Superinfection
		• Serious adverse event
		• Severe sepsis
		• Developed IV line infection
**Hospital characteristics**		• Discharge physician specialty, Type of hospital
		• Overall hospital beds,
		• Hospital has an ED protocol (IV-to-oral antibiotic switch or OPAT),
		• Hospital has an IV-to-oral antibiotic switch protocol

*Abbreviations:* cSSTI, complicated skin and soft tissue infection; IV, intravenous; ED, early discharge; ES, early switch; LOS, length of hospital stay; MRSA, methicillin-resistant *Staphylococcus aureus*; OPAT, outpatient parenteral antibiotic therapy. Add OPAT?

### Real-world healthcare resource use

Length of IV therapy, LOS, and IV-to-oral antibiotic switch measures were assessed using data abstracted from patients’ medical charts. This represented actual (observed) healthcare resource use. Length of IV therapy was defined as the time between the start of MRSA-active IV therapy and the last date of inpatient IV antibiotic use. LOS was measured from the date of hospital admission for patients who were admitted for treatment of MRSA cSSTI, or otherwise from the date of diagnosis of cSSTI (cSSTI index date) to the date of hospital discharge. IV-to-oral antibiotic switch included any inpatient who was switched from IV-to-oral antibiotic therapy before discharge from the hospital.

### Opportunities for early switch and early discharge

Opportunities for further reduction in length of IV therapy (ES) or LOS (ED) might exist for some patients. ES and ED criteria were developed through literature review [14–21] and expert consensus, and the date that patient met each of the criteria (if at all) determined retrospectively by the study investigator. ES eligibility required that the patient meet all of the following criteria prior to IV-to-oral switch or discontinuation: stable clinical infection; afebrile (i.e. temperature <38°C for 24 hours); normalized white blood cell count (i.e. not <4 X 10^9^/L or >12 X 10^9^/L); no unexplained tachycardia; systolic blood pressure ≥100 mm Hg; and tolerated oral fluids, diet, and medications with no gastrointestinal absorption problems. ED eligibility required the patient to meet all of the ES criteria prior to discharge and have no reason to remain hospitalized except for infection management.

### Statistical analysis

Pearson correlation coefficients were calculated between outcome variables (LOS, IV days, IV-to-PO switch, ES eligibility, ED eligibility) to determine their interrelationship. Following this, multivariable regression models were run for each outcome variable. Before modelling, clinical expert authors reviewed the list of potential variables to include in models and identified variables that were clinically important and as such should always be included within each multivariable model, as labeled in Table 1. Candidate variables with a p-value ≤0.1 within bivariate tests (analysis of variance, chi-squared, correlation) and variables that were clinically important were tested for inclusion within a series of statistical models including forward, backward, and stepwise selection models. Forward selection models forced the inclusion of all clinically important variables, but backwards and stepwise models allowed for removal of these variables. Models were compared based on their goodness of fit. In the event that no single model appeared to best fit the data, clinical authors were consulted regarding which model was most clinically relevant.

For length of IV therapy and LOS outcomes, ordinary least-squares linear regression analyses were conducted using the MRSA treatment sample and the full sample, respectively. For the IV-to-oral antibiotic switch outcome, logistic regressions used a subgroup of patients in the MRSA treatment sample who received either IV-only antibiotics (reference group) or IV-to-oral antibiotic switch therapy. For ES and ED eligibility outcomes, logistic regressions were run using the full sample. Only IV-to-oral antibiotic switch was considered as both an outcome in one model and a predictor in subsequent models.

For the purposes of analysis, patients from Ireland were combined with those from the United Kingdom for multivariable models, because of Ireland’s small sample size. The discharge physician’s specialty was used as a proxy for the treating physician’s specialty, as data on the latter were not collected. When present, missing data was treated as a separate category for independent variables. This most frequently occurred when information was not documented within patients’ charts.

## Results

### Study samples and demographic and clinical characteristics of the full sample

The full sample included 1542 patients, comprising 1502 patients randomly selected by 342 physicians/sites and an additional 40 oversampled patients who received IV-to-oral MRSA-active antibiotic switch therapy. Patients in the full sample were mean ± standard deviation aged 60.8 ± 16.5 years, with more males (61.5%) than females (38.5%), and the majority were white (92.9%; Table 2). Most had MRSA cSSTI as the primary cause of hospitalization (81.0%). Among the full sample, 1,508 patients received confirmed MRSA-active therapies, of whom 1,228 received IV-only therapy and 197 received IV-to-oral antibiotic switch therapy. These 2 groups (a total of 1,425 patients) were used in models of IV-to-oral antibiotic switch. If patients received both IV and oral medications in the hospital, but did not switch from IV-to-oral antibiotic therapy or were discharged on OPAT, they were excluded.

**Table 2:** **Patient demographic and clinical characteristics in the full sample**

Characteristic	Overall (N = 1542)
Mean ± SD age, years	60.8 ± 16.5
Male, n (%)	949 (61.5)
White, n (%)	1432 (92.9)
Mean ± SD CCI score	2.3 ± 2.2
Primary reason for hospitalization is treatment of MRSA cSSTI, n (%)	1249 (81.0)
Timing of cSSTI index date, n (%)	
At hospital admission	1282 (83.1)
1–3 days after admission	49 (3.2)
≥4 days after admission	211 (13.7)
Type of cSSTI, n (%)	
Surgical site infection or posttraumatic wound	400 (25.9)
Major abscess	271 (17.6)
Infected ulcer	381 (24.7)
Deep or extensive cellulitis	406 (26.3)
Other (including infected burn)	84 (5.4)
cSSTI location, n (%)	
Head/skull/neck	63 (4.1)
Torso/abdomen	325 (21.1)
Upper extremity	232 (15.0)
Lower extremity	922 (59.8)
Sepsis, severe sepsis, or septic shock during cSSTI episode, n (%)	265 (17.2)
Surgical procedures for treatment of cSSTI, n (%)	597 (38.7)
Mean ± SD number of procedures among patients with any procedures	0.4 ± 0.6
Patient switched from IV-to-oral inpatient MRSA-active antibiotic treatment, n (%)	197 (12.8)
Patient received MRSA-targeted therapy at discharge, n (%)	514 (33.3)

*Abbreviations:* CCI, Charlson comorbidity index; cSSTI, complicated skin and soft tissue infection; IV, intravenous; MRSA, methicillin-resistant *Staphylococcus aureus*; SD, standard deviation.

### Real-world healthcare resource use

A relatively strong positive correlation was found between length of IV therapy and LOS (r = 0.66), with a weaker negative correlation between length of IV therapy and IV-to-oral antibiotic switch (r = −0.18; Table 3).

**Table 3:** **Correlations between key outcomes**

	IV-to-oral antibiotic switch	Length of IV therapy	LOS	ES eligible	ED eligible
IV-to-oral antibiotic switch					
r value	1.00	−0.18	−0.03	−0.11	0.05
p value	N/A	<0.001	0.192	<0.001	0.073
Length of IV therapy					
r value	−0.18	1.00	0.66	0.11	0.03
p value	<0.001	N/A	<0.001	<0.001	0.200
LOS					
r value	−0.03	0.66	1.00	−0.05	0.02
p value	0.192	<0.001	N/A	0.053	0.482
ES eligible					
r value	−0.11	0.11	−0.05	1.00	0.44
p value	<0.001	<0.001	0.053	N/A	<0.001
ED eligible					
r value	0.05	0.03	0.02	0.44	1.00
p value	0.073	0.200	0.482	<0.001	N/A

*Abbreviations:* ED, early discharge; ES, early switch; IV, intravenous; LOS, length of hospital stay; N/A, not applicable.

Outcomes were evaluated by patient baseline characteristics and these outcomes varied across countries. After adjustment for covariates within multivariable models (Table 4), patients in Austria, Germany, Greece, Italy, Poland, and Portugal were significantly less likely to switch from IV-to-oral therapy compared with patients in the United Kingdom or Ireland (all p < 0.05, Table 4). In addition, the length of IV therapy was longer in France, Germany, Greece, Italy, Poland, Portugal, Slovakia, and Spain; LOS was also longer in all of these countries except Greece and Slovakia (all p < 0.05) (Additional file 1).

**Table 4:** **Significant covariates within final regression models for actual treatment patterns**

Level	IV-to-oral antibiotic switch (n = 1425)	Length of IV therapy (n = 1508)	LOS (n = 1542)
	OR (95% CI)	β (SE)	β (SE)
Intercept	–	5.98 (1.71)***	24.72 (2.47)***
**Patient baseline characteristics**			
Country (vs Ireland/United Kingdom)			
Austria	0.17 (0.04–0.76)*	2.74 (1.49)	2.48 (2.64)
France	1.44 (0.81–2.57)	4.24 (0.93)***	6.23 (1.66)***
Germany	0.40 (0.21–0.78)**	4.16 (0.97)***	4.28 (1.74)*
Greece	0.05 (0.01–0.18)***	2.53 (1.14)*	1.25 (2.03)
Italy	0.23 (0.10–0.55)***	2.91 (1.04)**	4.69 (1.87)*
Poland	0.19 (0.04–0.90)*	7.65 (1.69)***	5.97 (2.93)*
Portugal	0.16 (0.06–0.40)***	4.41 (1.18)***	4.46 (2.12)*
Slovakia	0.67 (0.23–1.93)	5.29 (1.83)**	3.32 (3.14)
Spain	0.90 (0.49–1.65)	3.10 (1.01)**	5.26 (1.81)**
IV drug abuse	0.72 (0.38–1.34)	2.78 (0.88)**	4.95 (1.72)**
CCI (continuous)	0.96 (0.89–1.04)	0.27 (0.12)*	0.44 (0.21)*
Any MRSA colonization before admission	1.77 (1.13–2.77)*		
Not reported/unknown	1.78 (1.19–2.67)**		
**Infection/treatment characteristics**			
cSSTI type (vs deep/extensive cellulitis)			
Surgical site infection or posttraumatic wound	1.26 (0.77–2.07)	−1.39 (0.70)*	−2.07 (1.24)
cSSTI location (vs torso/abdomen)			
Upper extremity	1.90 (1.09–3.33)*	−1.62 (0.80)*	−4.97 (1.43)***
Hospital-acquired or healthcare-associated infection unknown/undocumented	0.98 (0.60–1.59)	−2.40 (0.68)***	−2.21 (1.20)
Days from admission to cSSTI index date (vs cSSTI at admission)			
≥4 days after admission		1.13 (0.64)	5.57 (1.13)***
MRSA-targeted therapy patterns (vs IV-only)			
IV-to-oral antibiotic switch	–	−5.19 (0.74)***	−1.86 (1.32)
Discharged on OPAT	–	−2.64 (1.59)	−6.92 (2.85)*
No MRSA-active antibiotic	–	–	−7.52 (2.98)*
Initial antibiotic therapy was MRSA active (vs was not MRSA active)	0.41 (0.24 - 0.70)**	5.84 (1.01)***	
Time to initiating MRSA-active therapy (vs on or before cSSTI index date)			
1–2 days post cSSTI index date		−1.34 (0.39)***	−2.03 (0.66)**
≥3 days post cSSTI index date		2.20 (0.53)***	4.18 (0.80)***
Physician specialty (vs GP)^a^			
IM	2.26 (1.43–3.56)***	1.18 (0.64)	2.86 (1.13)*
Infectious disease	3.01 (1.88–4.82)***	2.39 (0.73)**	1.28 (1.30)
Surgeon	1.31 (0.70–2.45)	2.73 (0.87)**	5.78 (1.55)***
Any surgical procedures for cSSTI	1.46 (1.00–2.12)*	0.81 (0.54)	1.80 (0.96)
**Complications**			
Severe sepsis		1.93 (0.32)***	2.32 (0.59)***
Superinfection			3.32 (0.95)***
Developed IV line infection			
**Hospital characteristics**			
Overall hospital beds (vs ≥1000)			
10–249	2.37 (1.45–3.88)***	−2.17 (0.77)**	−2.17 (1.37)
Hospital had an IV-to-oral antibiotic switch protocol		0.88 (0.61)	−2.13 (1.08)*

*p < 0.05; **p < 0.01; ***p < 0.001.

^a^Discharge physician specialty was used as a proxy for treating physician specialty.

Full versions of the final models, including covariates that were statistically non-significant, are in Additional file 1: Table S1. Abbreviations: CCI, Charlson comorbidity index; CI, confidence interval; cSSTI, complicated skin and soft tissue infection; IV, intravenous; ED, early discharge; ES, early switch; GP, general practitioner; IM, internal medicine specialist; LOS, length of hospital stay; MRSA, methicillin-resistant *Staphylococcus aureus*; OPAT, outpatient parenteral antibiotic therapy; OR, odds ratio; SAE, serious adverse event; SE, standard error.

IV drug abuse was associated with significantly longer length of IV therapy (by 2.78 days) and LOS (by 4.95 days). Length of IV therapy and LOS also became longer with increasing CCI score, but CCI score did not significantly impact the likelihood of IV-to-oral antibiotic switch (Table 4). Patients with any MRSA colonization prior to admission were significantly more likely to be switched from IV-to-oral antibiotic therapy.

Certain infection characteristics were also associated with actual treatment patterns (Table 4). Compared with patients with deep or extensive cellulitis, patients with a surgical site or posttraumatic wound infection had significantly shorter length of IV therapy as well as 2.07 days shorter LOS (p > 0.05). Compared with patients whose infection was in the torso or abdomen, patients with an upper extremity infection were significantly more likely to switch from IV-to-oral therapy, had shorter length of IV therapy, and shorter LOS. Patients whose infection had developed ≥4 days after admission had longer LOS after diagnosis of infection.

In terms of treatment characteristics, IV-to-oral antibiotic switch was associated with 5.19 days shorter duration of IV therapy (p < 0.001) and 1.86 days shorter LOS (p > 0.05; Table 4). Patients who were discharged from the hospital and received outpatient parenteral antibiotics had numerically shorter inpatient length of IV therapy and significantly shorter LOS compared with those who received IV-only inpatient treatment. Patients who were not treated with a confirmed MRSA-active therapy also had a shorter LOS versus patients treated with IV-only therapy (p < 0.05). Patients whose initial antibiotic treatment was MRSA-active were significantly less likely to receive IV-to-oral antibiotic switch therapy compared with those who received no MRSA-active therapy and had longer lengths of IV therapy on average. Interestingly, patients who started MRSA-active treatment 1 or 2 days after their cSSTI index date had significantly shorter length of IV therapy (p < 0.001) and LOS (p < 0.01) compared with those who initiated MRSA-active therapy on or before the day their cSSTI was diagnosed; however, those who initiated therapy ≥3 days after their cSSTI was diagnosed had significantly longer lengths of IV therapy and LOS (both p < 0.001). Compared with patients treated by a general practice physician, patients who were treated by an internal medicine or infectious disease physician were more likely to be switched from IV-to-oral therapy. Patients treated by an infectious disease physician or surgeon had significantly longer length of IV therapy and patients treated by an internal medicine physician or surgeon had significantly longer LOS (all p < 0.05). Patients who had (vs those without) any surgical procedure for their cSSTI were also significantly more likely to be switched from IV-to-oral therapy, but also had numerically longer length of IV therapy and LOS.

Length of IV therapy and LOS patterns may have also been driven by certain infection complications (Table 4). For example, patients who developed severe sepsis had significantly longer length of IV therapy and LOS; patients who developed a superinfection also had significantly longer LOS.

When hospital characteristics were considered (Table 4), patients treated in smaller hospitals (10–249 bed capacities compared with ≥1000 beds) were significantly more likely to be switched from IV-to-oral therapy, had approximately 2 days shorter length of IV therapy and numerically but not statistically significantly shorter LOS. Patients treated in a hospital with an IV-to-oral antibiotic switch protocol also were discharged 2 days earlier on average (p < 0.05).

### Opportunities for early switch and early discharge

A relatively strong positive correlation was found between eligibilities for ES and ED (r = 0.44). Although correlations between ES and resource use (i.e. length of IV therapy and IV-to-oral antibiotic switch) reached statistical significance (p < 0.05), the correlations were generally negligible (Table 3).

Similar to the results for treatment patterns, variability was found in the odds of ES and ED eligibility across countries, with significantly greater odds (compared with the UK and Ireland) of ES eligibility in Germany, Greece, and Portugal and greater odds of ED eligibility in the Czech Republic and Germany, but reduced odds in Slovakia (Table 5). Patients’ living arrangements significantly impacted ED eligibility, with patients living alone without a caregiver being significantly more likely to meet ED criteria compared with those living at home with a caregiver. Patients with comorbid dementia were also significantly less likely to be ES and ED eligible (both p < 0.01).

**Table 5:** **Significant covariates within final regression models for ES and ED eligibility opportunities**

Level	ES eligibility (n = 1542)	ED eligibility (n = 1542)
	OR (95% CI)	OR (95% CI)
Intercept		
**Patient baseline characteristics**		
Country (vs Ireland/United Kingdom)		
Czech Republic	0.91 (0.38–2.17)	2.18 (1.01–4.67)*
Germany	2.47 (1.55–3.92)***	2.05 (1.30–3.24)**
Greece	3.30 (1.99–5.48)***	1.39 (0.84–2.30)
Portugal	1.75 (1.04–2.95)*	1.67 (0.99–2.83)
Slovakia	0.55 (0.20–1.46)	0.29 (0.10–0.82)*
Patient living arrangements (vs at home with caregiver)		
Alone without caregiver		1.33 (1.03–1.72)*
Dementia	0.46 (0.28–0.76)**	0.50 (0.30–0.83)**
**Infection/ treatment characteristics**		
Hospital-acquired or healthcare-associated infection	1.75 (1.30–2.37)***	1.69 (1.25–2.27)***
Days to first MRSA culture (vs on or before cSSTI index date)		
No MRSA culture documented	0.46 (0.30–0.69)***	0.58 (0.40–0.85)**
Days from admission to cSSTI index date (vs cSSTI at admission)		
≥4 days after admission	0.47 (0.32–0.70)*	0.55 (0.38–0.79)
MRSA-targeted therapy patterns (vs IV-only)		
IV-to-oral antibiotic switch	0.49 (0.33–0.72)***	1.40 (1.00–1.96)
Time to initiating MRSA-active therapy (vs on or before cSSTI index date)		
1–2 days post cSSTI index date		1.20 (0.92–1.56)**
≥3 days post cSSTI index date		0.71 (0.51–0.99)**
Any surgical procedures for cSSTI	1.13 (0.87–1.45)	1.04 (0.82–1.32)
**Complications**		
Developed IV line infection	0.19 (0.06–0.56)**	
**Hospital characteristics**		
Hospital had an IV-to-oral antibiotic switch protocol		0.58 (0.43–0.80)***
Hospital had an ED protocol (IV-to-oral antibiotic switch or OPAT)		1.86 (1.31–2.64)***

*p < 0.05; **p < 0.01; ***p < 0.001.

Full versions of the final models, including covariates that were statistically non-significant, are in Appendix Table 1.

Abbreviations: CCI, Charlson comorbidity index; CI, confidence interval; cSSTI, complicated skin and soft tissue infection; IV, intravenous; ED, early discharge; ES, early switch; GP, general practitioner; IM, internal medicine specialist; LOS, length of hospital stay; MRSA, methicillin-resistant *Staphylococcus aureus*; OPAT, outpatient parenteral antibiotic therapy; SAE, serious adverse event; SE, standard error.

When considering infection characteristics (Table 5), cSSTI type and location did not appear to have a significant impact on ES or ED eligibility. However, patients with a hospital-acquired or healthcare-associated MRSA cSSTI were significantly more likely to meet ES and ED criteria (both p < 0.001). Patients without a documented MRSA culture were also significantly less likely to meet ES criteria and ED criteria compared with patients with first MRSA culture on or before the cSSTI index date.

The amount of time to initiation of MRSA-active therapy impacted ED eligibility, but not ES eligibility (Table 5). Compared with patients who started MRSA-active therapy on or before their cSSTI index date, those who began MRSA-active therapy 1 to 2 days post cSSTI index date had increased odds of ED eligibility (p < 0.01) whereas those who started therapy ≥3 days post cSSTI index date had a decreased likelihood of ED eligibility (p < 0.01), mirroring the LOS results. The odds of ES were reduced in patients with ≥4 days from admission to cSSTI index date compared with patients who had their cSSTI index date at admission.In relation tocomplications (Table 5), patients who developed an IV line infection were significantly less likely to be ES eligible (p < 0.01).

When considering hospital protocols, patients treated in hospitals that had an IV-to-oral antibiotic switch protocol were less likely to be ED eligible, while patients treated in hospitals that had an ED protocol were more likely to be ED eligible (p < 0.001).

## Discussion and conclusions

This analysis highlights the various predictors of IV-to-oral antibiotic switch, length of IV therapy, LOS, and opportunities for ES and ED within a selected European MRSA cSSTI cohort. Both actual practice patterns (i.e. IV-to-oral antibiotic switch rates, length of IV therapy, LOS) and ES and ED eligibility varied significantly across countries both in bivariate and multivariable analyses. After adjustment for key clinical and demographic characteristics, actual length of IV therapy and LOS were lower for patients who received IV-to-oral switch MRSA-active antibiotic, but the difference was not statistically significant for LOS. Patients who received IV-to-oral antibiotic switch therapy appeared to be more likely to be ED eligible compared with patients treated with IV-only therapy, although the difference was not statistically significant.

In reviewing the specific predictors of each outcome, it should be noted that IV-to-oral antibiotic switch patients represent those for whom resource use has likely been optimized in terms of length of IV therapy. Likewise, some of the patients who were not ED eligible were only not eligible because they were discharged on the day that all criteria were met (i.e., discharge was optimized). For this reason, it is not surprising that a number of variables were significant predictors for actual practice patterns but they did not significantly predict ES and ED eligibility. For example, patients with MRSA cSSTI located in the upper extremities were more likely to switch from IV-to-oral antibiotics, had fewer days of IV therapy, and shorter LOS, but did not have a higher or lower likelihood of ES/ED eligibility. This highlights an important difference between length of IV therapy, LOS, and IV-to-oral antibiotic switch outcomes versus ES and ED eligibility: practice patterns reflect what actually happened, while ES and ED eligibility suggest hypothetical opportunities for improving care. Given this distinction, these outcomes should plausibly have different key predictors.

The importance of early treatment for cSSTI was underscored by patients who received therapy 1 or 2 days after their cSSTI index date having significantly lower length of IV therapy and LOS, with increased odds of ED eligibility. In contrast, in patients whose treatment for cSSTI occurred ≥3 days after their cSSTI index date, the length of IV therapy and LOS were significantly longer, with decreased odds of ED eligibility.

A number of patient- and hospital-level predictors were found to influence directionality of actual resource utilization. Treatment in small hospitals (<250 beds) compared with larger facilities (≥1000 beds), for example, was predictive of IV-to-oral antibiotic switch and fewer IV line days. One potential explanation for this trend may be that smaller facilities are motivated by limited resources to minimize IV therapy when possible. Conversely, patients treated by certain clinical specialties were associated with significantly longer length of IV therapy (i.e. infectious disease physicians or surgeons) and LOS (i.e. internists or surgeons). This result could be related to treating physicians’ clinical disciplines, with specialists being more likely to treat the more severe cSSTI cases compared with non-specialists. However, information was only available about the discharge physicians’ specialties, which were used as a proxy for the treating physicians’ clinical specialties.

Hospitals with an established IV-to-oral antibiotic switch protocol had lower odds of ED eligibility, but were also associated with 2.13 fewer days in LOS, which could potentially result in cost savings associated with fewer bed days. Hospitals with ED protocols had nearly double the odds (OR, 1.86) of patients achieving ED eligibility, which could indicate that these programs are using different criteria to identify patients who are eligible for ED, or that they are not fully implemented. One limitation to note was that full information regarding the structure of IV-to-oral antibiotic switch and ED protocols at the site level were not collected; thus, any conclusions regarding the impact of these protocols must be interpreted with care.

Our results can be compared to those observed in other similar studies, although these studies were smaller and represent a diverse range of infections and healthcare systems. Dryden *et al*. and Gray *et al.* identified patients eligible for ES and ED using an evaluation audit tool to assess patients receiving antibiotic treatment in acute medical and surgical wards across six centres in the UK [22, 23]. Criteria utilized evaluated the duration of antibiotic therapy, the patient’s ability to tolerate PO therapy, the presence of sepsis syndrome, signs and symptoms indicating infection resolution and the presence of comorbidities and social factors that could influence hospital discharge. A total of 34% of patients requiring antibiotic therapy were eligible for PO therapy; 21% of patients were eligible for discharge. Ten patients required OPAT, 55 patients required PO antibiotic therapy and 24 patients required no therapy. Factors that appeared to prevent discharge were many and included waiting for nursing home placement, requiring social services or rehabilitation, presence of comorbidities and requiring further medical or surgical input. These factors are particularly relevant to elderly patients in acute care. Indeed, another study identified deconditioning, on-going infection, social issues and cardiovascular disorders as key factors preventing discharge [24].

Oral antibiotic administration also enables optimization of inpatient bed use. Between the years 1998 and 2008 hospitals in 15 European countries saw a mean decrease of 18% in acute care hospital bed capacity per 100, 000 population [9]. During this same time period in 12 European countries there was an average reduction in hospital length of stay (LOS) of 2 days, resulting in a mean hospital LOS of 6.5 days [9]. A decrease in both acute care hospital bed capacity and hospital LOS may indicate a significant pressure on inpatient bed capacity [9]. This reduction in inpatient capacity and stay is driven primarily by changing technology, such as diagnostic and treatment procedures or techniques that allow patients to be managed in alternative settings, and availability of more effective treatments [25]. In infectious diseases practice, progress was made in the development of effective PO treatments for serious infections, although there is still substantial growth potential [26].

The results also highlight some of the barriers against optimizing care, including social situations and healthcare infrastructure. For example, patients with history of IV drug abuse had longer lengths of IV treatment and LOS, as did patients with multiple comorbidities. Patients with dementia, not surprisingly, had lower odds of ES/ED eligibility. These complex patients represent challenges in streamlining hospital discharge; however, in more straightforward patient situations, our study suggests additional opportunities may optimize efficiency of care. While patient complexity is one barrier, the healthcare infrastructure also plays a role in optimizing care, as patients discharged on OPAT had much shorter LOS; however, not all areas in Europe have well-established OPAT programs.

This study was conducted as a retrospective medical chart review and therefore the results were limited by the completeness of the information that was recorded in those charts and data that was collected. Of note, patient preferences, such as the desire to be discharged sooner, would likely impact actual practice patterns but unfortunately are not available through chart documentation. While presence of bacteremia secondary to the presence of the cSSTI may have also been a mediating factor potentially increasing resource use, this was not collected and severe sepsis could serve as a surrogate marker. Likewise we used discharge physician specialty as a surrogate marker of treating physician specialty in the absence of this data point.

A large number of patients with documented MRSA cSSTI were enrolled from 342 sites across 12 European countries, which provided useful real-world data to determine potential savings in both length of IV therapy and hospital bed days. However, patients were not equally distributed in all of the 12 countries included in this analysis, with ~70% of them from the following countries: France, Germany, Italy, Spain, and the United Kingdom. Nevertheless, this patient distribution may reflect European MRSA cSSTI epidemiology, since the countries with fewer patients in these analyses (e.g. Austria, Slovakia, Poland, Czech Republic) had published reporting MRSA rates of 7% to 14% compared with at least 25% in the 5 Western European countries mentioned above [27].

In conclusion, actual healthcare resource use and ES and ED eligibility varied significantly across countries both in bivariate and multivariable analyses. The association of IV-to-oral antibiotic switch therapy with shorter length of IV therapy days and LOS (though not significant) indicates that switching patients may be associated with reduced resource use and hence cost savings and benefits to patients. Rates of ES and ED eligibility also indicate that many opportunities are available to optimize actual practice patterns, particularly through the identification and targeting of interventions to patient populations with greater eligibility and longer LOS and IV days. These real world data are multifaceted and can be challenging in their collection and interpretation. However, this unique study provides useful insights into the dynamics of everyday decision making when managing these patients and we hope these findings will encourage hospitals across Europe to examine their systems and processes of care in relation to ES and ED for this and other therapeutic areas. The fiscal and quality of care benefits could be potentially great.

## Electronic supplementary material

Additional file 1: Table S1: Results of the final regression models, by outcome. (XLSX 21 KB)

## Abbreviations

CI: Confidence interval
CCI: Charlson comorbidity index
cSSTI: Complicated skin and soft tissue infection
ED: Early discharge
ES: Early switch
IV: Intravenous
LOS: Length of stay
MRSA: Methicillin-resistant *Staphylococcus aureus*
OPAT: Outpatient parenteral antibiotic therapy
OR: Odds ratio.
